# Transarterial chemoembolization plus sorafenib for the management of unresectable hepatocellular carcinoma: a systematic review and meta-analysis

**DOI:** 10.1186/s12876-018-0849-0

**Published:** 2018-09-04

**Authors:** Lin Li, Wenzhuo Zhao, Mengmeng Wang, Jie Hu, Enxin Wang, Yan Zhao, Lei Liu

**Affiliations:** 1 Department of Gastroenterology, Tangdu Hospital, Military Medical University of PLA Airforce (Fourth Military Medical University), 1 Xinsi Road, Xi’an, 710038 China; 2 Department of Drug and Equipment, Aeromedicine Identification and Training Centre of Air Force, Lintong District, Xi’an, China; 3 Department of Liver Disease and Digestive Interventional Radiology, Xijing Hospital of Digestive Diseases, Military Medical University of PLA Airforce (Fourth Military Medical University), Xi’an, China; 4 Department of Gastroenterology, First Affiliated Hospital of Xi’an Jiaotong University, 277 West Yanta Road, Xi’an, 710061 China; 5 Cell Engineering Research Center and Department of Cell Biology, State Key Laboratory of Cancer Biology, Military Medical University of PLA Airforce), Xi’an, China

**Keywords:** Hepatocellular carcinoma, Transarterial chemoembolization, Sorafenib, Systemic review, Meta-analysis

## Abstract

**Background:**

Transarterial chemoembolization (TACE) is the recommended treatment for hepatocellular carcinoma (HCC) patients at Barcelona Clinic Liver Cancer (BCLC) B-stage, whereas sorafenib is an orally administered small molecule target drug for BCLC C-stage. This updated systemic review and meta-analysis focuses on identifying the efficacy of the combination of TACE with sorafenib, which remains controversial despite years of exploration.

**Methods:**

PubMed, EMBASE, Scopus and the Cochrane Library were systematically reviewed to search for studies published from January 1990 to May 2017. Studies focusing on the efficacy of combination therapy for unresectable HCC were eligible. The hazard ratio (HR) with 95% confidence intervals (95% CIs) for time to progression (TTP), overall survival (OS), disease control rate (DCR) and aetiology were collected. The data were then analysed through fixed/random effects meta-analysis models with STATA 13.0. The incidence and severity of treatment-related adverse events (AEs) were also evaluated.

**Results:**

Twenty-seven studies were included. Thirteen non-comparative studies reported median OS (ranging from 18.5 to 20.4 months), median TTP (ranging from 7 to 13.9 months) and DCR (ranging from 18.4 to 95%). Fourteen comparative studies provided median OS (ranging from 7.0 to 29.7 months) and median TTP (ranging from 2.6 to 10.2 months). Five comparative studies provided DCR (ranging from 32 to 97.2%). Forest plots showed that combination therapy significantly improved TTP (HR = 0.66, 95% CI 0.50–0.81, *P* = 0.002) rather than OS (HR = 0.63, 95% CI 0.55–0.71, *P* = 0.058), compared to TACE alone. DCR increased significantly in the combination therapy group (OR = 2.93, 95% CI 1.59–5.41, *P* = 0.005). Additional forest plots were drawn and no significant differences were observed with regard to survival outcome among various aetiologies. Forest plots for separate analysis of regions showed the HR for TTP was 0.62 (95% CI 0.45–0.79, *P* = 0.002) in the Asian countries group, and 0.82 (95% CI 0.59–1.05, *P* = 0.504)) in western countries. The HR for OS was 0.61 (95% CI 0.48–0.75, *P* = 0.050) in the Asian countries group and was 0.88 (95% CI 0.56–1.20, *P* = 0.845) in western countries. These data may indicate positive TTP outcome in Asian patients but not in European patients while no positive findings regarding OS were observed in either region. The most common AEs included fatigue, hand-foot skin reaction, diarrhoea and hypertension.

**Conclusions:**

Combination therapy may benefit unresectable HCC patients in terms of prolonged TTP and DCR. More well-designed studies are needed to investigate its superiority for OS.

**Electronic supplementary material:**

The online version of this article (10.1186/s12876-018-0849-0) contains supplementary material, which is available to authorized users.

## Background

Hepatocellular carcinoma (HCC) is the most common liver malignancy. Causing approximate 700,000 deaths per year around the world, it is the third leading cause of cancer death and the fifth most common malignancy globally [1]. Furthermore, Asian countries contribute a large proportion of global HCC, making it a heavy burden in the Asia-Pacific region [2].

Currently, the most widely perceived staging system for HCC is the Barcelona Clinic Liver Cancer (BCLC) system, which integrates prognostic classification and corresponding treatment of HCC. According to the BCLC system, very early and early-stage HCC (BCLC 0 or A) should be treated with curative modalities [3–5], whereas BCLC B and C HCC classified as unresectable HCC should be considered for transarterial chemoembolization (TACE) and sorafenib, respectively [1].

Previous randomized controlled trials (RCTs) have shown that TACE can bring survival benefits to unresectable HCC patients [1]. However, the high recurrence rate after TACE treatment is a major limitation of conventional TACE (c-TACE), possibly resulting from increased expression of vascular endothelial growth factor (VEGF) and vplatelet-derived growth factor (PDGF). Repeated TACE may cause liver function deterioration [6]. Fortunately, as an inhibitor of many kinases, sorafenib can reduce proliferation and angiogenesis of tumour cells, increasing tumour apoptosis by inhibiting VEGF and PDGF receptors [7]. Therefore, combining sorafenib with TACE may be a promising strategy to reduce the recurrence rate of disease and improve the treatment efficacy compared to TACE mono-therapy [2].

Several clinical trials have evaluated survival outcomes in HCC patients who received combination therapy, but the findings differed greatly among studies and thus remain debatable. It remains a pending issue as to whether TACE plus sorafenib enhances TACE efficacy and improves survival. This updated meta-analysis aimed to analyse relevant clinical trials in recent years as much as possible (including comparative and non-comparative trials) to evaluate the efficacy of combination therapy used for unresectable HCC patients and ascertain the benefits of combination therapy.

## Methods

### Identification and eligibility of relevant studies

To cover as much of the relevant literature as possible, we comprehensively searched PubMed, EMBASE, Scopus and the Cochrane Library for studies published from January 1990 to May 2017. Search terms were as follows: “transarterial chemoembolization” or “chemoembolization” or “TACE” AND “hepatocellular carcinoma” or “hematoma” or “HCC” or “liver cancer” or “liver tumour” AND “sorafenib”. The references of retrieved articles were also screened. The search was limited to English articles involving only adult patients.

### Inclusion and exclusion criteria

#### Inclusion criteria

Studies that focused on combination therapy of sorafenib plus TACE in unresectable HCC were included. Studies were limited to English articles and adult patients. Necessary information included overall survival (OS), time to progression (TTP), disease control rate (DCR), adverse events (AEs) and tumour response.

#### Exclusion criteria

Studies that compared efficacy of combination therapy versus sorafenib alone were excluded. Non-English studies or comments, editorials, letters, case reports, reviews and meta-analyses were not considered. Studies unrelated to our topic or lacking useful information were also excluded.

### Definitions and standardization

Two types of TACE were analysed in our meta-analysis, including conventional TACE (c-TACE) and TACE with drug-eluting beads (DEB-TACE). Treatments including TACE before or after sorafenib were both defined as combination therapies. Patients should receive at least one session of TACE during their treatment.

TTP was defined as the time from initial treatment to tumour progression or last follow-up. OS was defined as the time from first TACE to the date of death or last follow-up. DCR was defined as the combination of complete response rate, partial response rate and stable disease rate.

### Data extraction

After initial identification of articles from databases, two researchers (Lin Li, Wenzhuo Zhao) screened studies according to the abovementioned criteria by reading titles and abstracts. At each screening step, the number of studies and the reasons for exclusion were recorded. Subsequently, the full-text of articles eligible for inclusion were independently assessed and necessary information was extracted, including baseline characteristics, treatment strategy, OS, TTP, DCR, AEs, HR and tumour responses. Finally, all available data were pooled and analysed. Disagreements between the two researchers were discussed until consensus was reached.

### Statistical analysis

Meta-analysis was performed by STATA 13.0 according to the Cochrane Handbook for Systematic Reviews of Interventions. The quality of included RCT studies was assessed by the Jadad scale [8], while non-RCT studies were assessed by the methodological index for non-randomized studies (MINORS) [9]. HR and 95% CI of TTP, OS, DCR, as well as aetiology of various studies were collected. I^2^ analysis was used to assess the heterogeneity among studies. If the I^2^ value was less than 50%, a fixed-effects meta-analysis model was conducted, and if the I^2^ value was not less than 50% the random-effects meta-analysis model was performed. For all outcomes, a *P*-value less than 0.05 was considered statistically significant.

## Results

### Identification of eligible studies

**Fig. 1:**
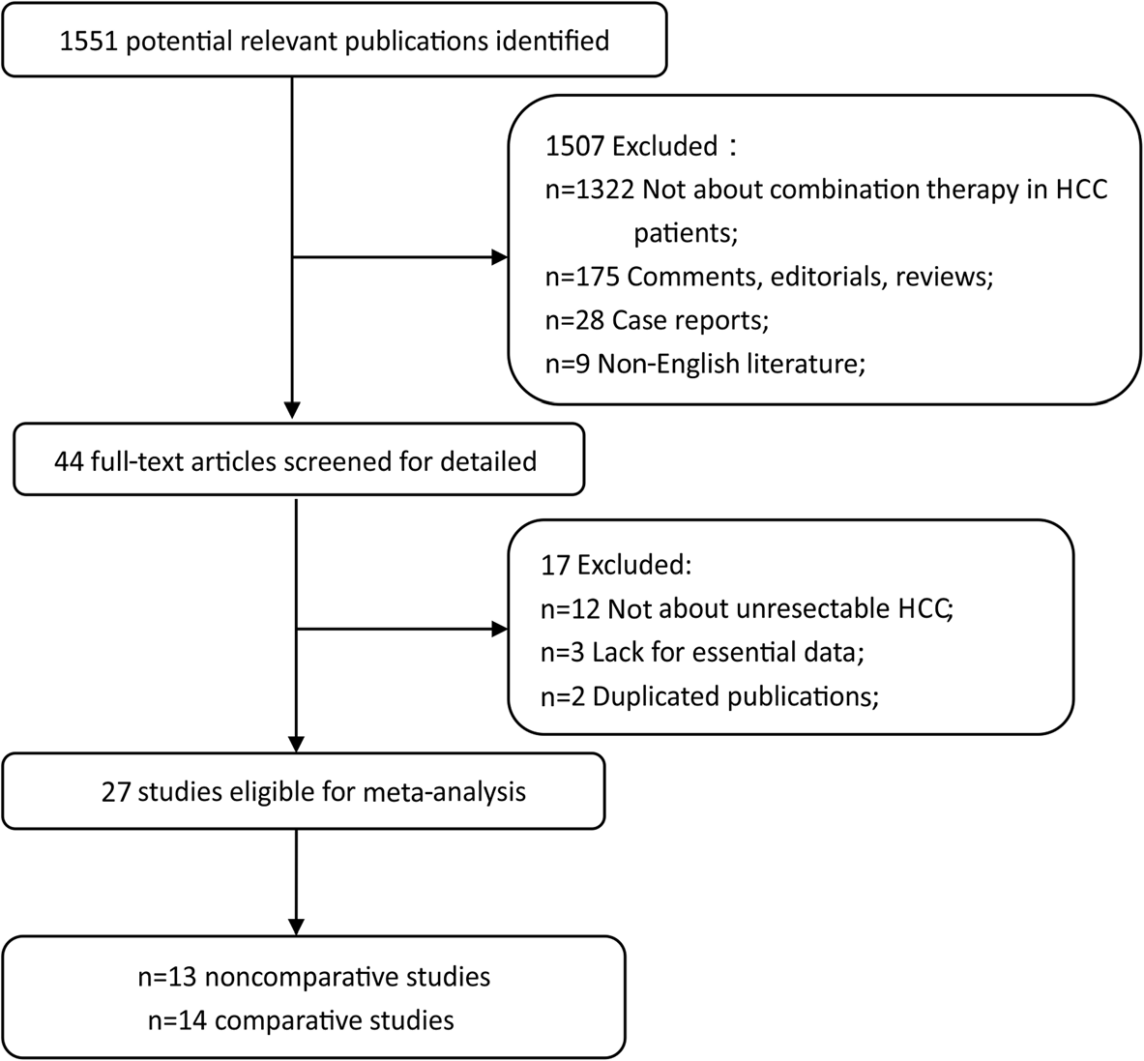
The study recruitment flowchart

After searching the literature within several databases, a total of 1551 studies were eventually identified for screening. According to titles and abstracts, 1507 studies were excluded, and the full texts of the remaining 44 articles were examined. Finally, 27 studies were included in our analysis, with 14 comparative studies and 13 non-comparative studies. The screening flowchart of the study is shown in Fig. 1.

### Study characteristics

The 13 non-comparative studies published from 2009 to 2016 included 8 phase-II studies, 2 phase-I studies and 3 retrospective studies (Table 1). C-TACE was used in 9 studies, and DEB-TACE was used in 4 studies. Seven of the thirteen studies were conducted in Asia. The number of patients per study ranged from 14 to 222. All patients in 13 non-comparative studies were graded as either Child-Pugh (CP) class A or B, among which most patients (65–94%) were at CP A. The proportion of patients at BCLC B stage was 20–100% and there were 1.9–80% at BCLC C stage. The ECOG performance status was reported to be 0 or 1 (94–100%). Eleven studies provided aetiology information about the patients. The total rates of hepatitis viral infection ranged from 24 to 100%. The detailed baseline characteristics of patients, duration of sorafenib and the number of TACE sessions (ranging from 1 to 3) are displayed in Table 1.

**Table 1:** Baseline characteristics of 13 non-comparative studies and patients

Authors (year) [Ref]	Study Design	Region	Patients	CPS	BCLC	ECOG	Aetiology	Treatment	No. of TACE	Duration Time of sorafenib
Erhardt et al. (2014) [33]	Phase II	Germany	38	≤8scores	NA	0–2	NA	Continuous sorafenib, interrupted only around TACE	2.0(mean)	NA
Dufour et al. (2010) [34]	Phase I Open-label	Switzerland	14	A = 93% B = 7%	B = 64% C = 36%	0 = 93% 1 = 7%	HCV = 29%	Sorafenib started 1 week prior to TACE without pause for TACE	2.0(median)	NA
Cabrera et al. (2011) ^a^ [35]	Phase II Prospective	USA	47	A = 72% B = 28%	B = 81% C = 19%	0 = 75% 1 = 25%	HCV = 60%	Continuous sorafenib started 2–4 weeks before DEB-TACE	3.0(median)	NA
Lee et al. (2011) [36]	Phase II Prospective	South Korea	59	A = 93% B = 7%	B = 100%	NA	HBV = 88%	Sorafenib, TACE was performed at every 6–8 weeks	NA	NA
Pawlik et al. (2011) ^a^ [37]	Phase II Prospective	USA	35	A = 89% B = 11%	B = 34% C = 66%	0 = 46% 1 = 54%	HCV = 37%	Continuous sorafenib started 1 week before DEB-TACE	2.0(median)	NA
Park et al. (2012) [38]	Phase II Prospective	South Korea	50	A = 94% B = 6%	B = 82% C = 18%	0 = 44% 1 = 56%	HBV = 68% HCV = 18%	Sorafenib started 3 days after TACE	1.0(median)	6 month
Sieghart et al. (2012) [39]	Phase I	Austria	15	A = 80% B = 20%	B = 70% C = 30%	0 = 92% 1 = 8%	HBV = 4% HCV = 20%	Sorafenib started 2 weeks before the first TACE	3.0(median)	5.2 month (median)
Chung et al. (2013) [40]	Phase II Prospective	China and South Korea	151	A = 92% B = 8%	A = 16% B = 82% C = 1.9%	0 = 82% 1 = 18%	NA	Sorafenib started 4–7 days after TACE	2.1(mean)	NA
Zhao et al. (2013) [41]	Prospective	China	222	A = 86% B = 14%	B = 20% C = 80%	0 = 44% 1 = 50% 2 = 6%	HBV = 80% HCV = 5%	Continuous sorafenib with no breaks before or after TACE	2.0(median)	NA
Pan et al. (2014) [7]	Retrospective	China	41	A = 85.4% B = 14.6%	NA	0 = 48.8% 1 = 51.2%	HBV = 97.6% HCV = 2.4%	Sorafenib was taken 3 days after the first TACE procedure	2.0(median)	NA
Chao et al. (2014) [2]	Phase II Prospective	Taiwan	192	A = 91.8% B = 7.1%	A = 16.9% B = 81.5% C = 1.6%	0 = 81.8% 1 = 17.7% 3 = 0.5%	NA	Sorafenib on day 4 (to day 7) after the first TACE (day 1) the interrupt on day 4 before the next TACE	3.0(median)	NA
Yao et al. (2015) [32]	Retrospective	China	50	A = 88% B = 12%	B = 52% C = 48%	0 = 46% 1 = 54%	HBV = 84% HCV = 4%	Sorafenib before and after 1 week of TACE	3.0(median)	1.4 month (median)
Cosgrove et al. (2015) ^a^ [42]	Phase II	USA	50	A = 92% B = 8%	A = 6% B = 32% C = 62%	0 = 52% 1 = 48%	HBV = 8% HCV = 44%	Sorafenib was started 1 week before the first round of DEB-TACE	2.0(median)	1.5 month

*Abbreviations*: *BCLC* The Barcelona Clinic Liver Cancer, *CPS* Child-Pugh classification, *ECOG* Eastern Cooperative Oncology Group, *NO*. number, *NA* not available, *HBV* hepatitis B virus, *HCV* hepatitis C virus

^a^ TACE with drug-eluting beads (DEB) was performed in the studies. Patients in other studies treated with conventional TACE (c-TACE)

Fourteen comparative studies enrolled 1689 patients in total, including 3 RCTs, 4 non-randomized controlled studies and 7 retrospective studies (Table 2). C-TACE was used in 11 studies and DEB-TACE was used in 3 studies. The proportions of patients at BCLC B and C stages were 15–100% and 38–100%, respectively. The ECOG Performance Status was 0 or 1 (71–100%). For aetiology, HBV (hepatitis B virus)/HCV (hepatitis C virus) infection rates varied greatly. Patients in the Asian-Pacific region were mostly infected with HBV, while Japanese and European countries had more HCV infections. In 13 comparative studies, patients were TACE-responsive before sorafenib administration. Some patients in the study of Ohki et al. were unresponsive to TACE. Detailed procedures in treatment of each study are also provided in Table 2.

**Table 2:** Baseline characteristics of 14 comparative studies and patients

Authors (year) [Ref]	Study Design	Region	Patients	CPS	BCLC	ECOG	Aetiology	Treatment	Quality Assessment
Martin et al. (2010) ^a^ [43]	Prospective	several countries	150	ST:B = 31% DT:B = 39%	NA	NA	NA	ST, *n* = 30; DT, *n* = 120.	17
Kudo et al. (2011) [15]	Phase III Randomized	Japan	229	A = 100%	NA	0 = 87% 1 = 13%	HBV = 20% HCV = 60%	Sorafenib was given 1–3 months after TACE till progression	18
		South Korea							
Sansonno et al. (2012) [44]	Phase II prospective randomized	Italy	40	A = 100%	B = 100%	0 = 86% 1 = 24%	HCV = 100%	Sorafenib started 1 month after TACE till progression nor unacceptable toxicity	4
Lencioni et al. (2012) ^a^ [10]	Phase II prospective randomized	several countries	307	A = 100%	B = 100%	0 = 100%	NA	Continuous sorafenib 3–7d before TACE	4
Qu et al. (2012) [45]	Retrospective	China	45	A = 65% B = 35%	B = 35% C = 65%	0 = 95% 1 = 5%	HBV = 100%	Sorafenib started after TACE	17
Bai et al. (2013) [46]	Prospective	China	82	A = 77% B = 23%	B = 23% C = 77%	0 = 36.5% 1 = 46.5%	HBV = 87.9% HCV = 4.9%	Continuous sorafenib started within 14d after TACE	19
						2 = 14.6%			
						3 = 1.2%			
						4 = 1.2%			
Muhammad et al. (2013) ^a^ [47]	Retrospective	USA	43	ST:A = 85% DT:A = 77%	A = 46% B = 15% C = 38%	NA	ST:HCV = 69% DT:HCV = 93%	Sorafenib started with 200 mg bid and then increased to 400 mg in the majority of patients	20
Huang et al. (2013) [48]	Prospective	China	155	NA	NA	NA	NA	Sorafenib started within 2 weeks of the first cycle of TACE	14
Hu et al. (2014) [14]	Retrospective	China	280	ST:A = 70.7% T:A = 67.7%	B = 100%	NA	ST:HBV = 82.9% T:HBV = 79.8%	Sorafenib after TACE	20
Ohki et al. (2015) [6]	Retrospective	Japan	95	ST:A = 70.8% T:A = 56.3%	NA	NA	ST:HCV = 75.0% T:HCV = 67.6%	Sorafenib was started within 2 weeks after TACE	17
Yao et al. (2016) [12]	Prospective	China	150	A = 84% B = 16%	B = 42% C = 58%	0 = 42% 1 = 58%	ST:HBV = 84% T:HBV = 83%	Sorafenib therapy was initiated within 1 week before or after the initial TACE treatment	20
Zhang et al. (2016) [49]	Retrospective	China	20	A = 100%	NA	0 = 85% 1 = 15%	HBV = 80%	Sorafenib was given with an interval of 4-7 days before or after TACE session	19
Wan et al. (2016) [50]	Retrospective	China	450	A = 87% B = 13%	NA	0–1 = 91% 2 = 9%	NA	Oral sorafenib was administrated before or after TACE	14
Varghese et al. (2017) [13]	Retrospective	India	124	B:A = 55.9% B = 44.1% C:A = 46.2% B = 53.8%	B = 47.6% C = 52.4%	NA	B:HBV = 37.3% HCV = 18.7% C:HBV = 26.2% HCV = 23%	Sorafenib was introduced 5d after TACE	17

*Abbreviations*: *BCLC* The Barcelona Clinic Liver Cancer, *CPS* Child-Pugh classification, *ECOG* Eastern Cooperative Oncology Group, *NA* not available, *ST* sorafenib plus TACE, *DT* DEB –TACE, *HBV* hepatitis B virus, *HCV* hepatitis C virus, *MINORS* methodological index for non-randomized studies

^a^ TACE with drug-eluting beads (DEB) was performed in the studies. Patients in other studies treated with conventional TACE (c-TACE). Quality assessment of RCT trial adopted Jadad scale. Scores of non-randomized experimental study were assessed by MINORS

### Tumour response, DCR, TTP, OS

#### Non-comparative studies

In terms of the assessment of tumour response, six studies applied the response evaluation in solid tumours (RECIST) and 6 studies applied the modified RECIST (mRECIST). Eleven studies reported DCR ranging from 18.4 to 95%. Six studies reported median TTP ranging from 7 to 13.9 months. Four studies reported median OS ranging from 12 to 20.4 months (Additional file 1: Table S1).

#### Comparative studies

##### DCR

In 14 comparative studies, five studies reported DCR in combined groups ranging from 32 to 97.2% (Additional file 2: Table S2). For all five studies, DCR in the combination therapy group was substantially higher than those in the TACE alone group. The forest plot showed that the increase of DCR in combination therapy was significant (OR = 2.93, 95% CI 1.59–5.41, *P* = 0.005).

##### TTP

**Fig. 2:**
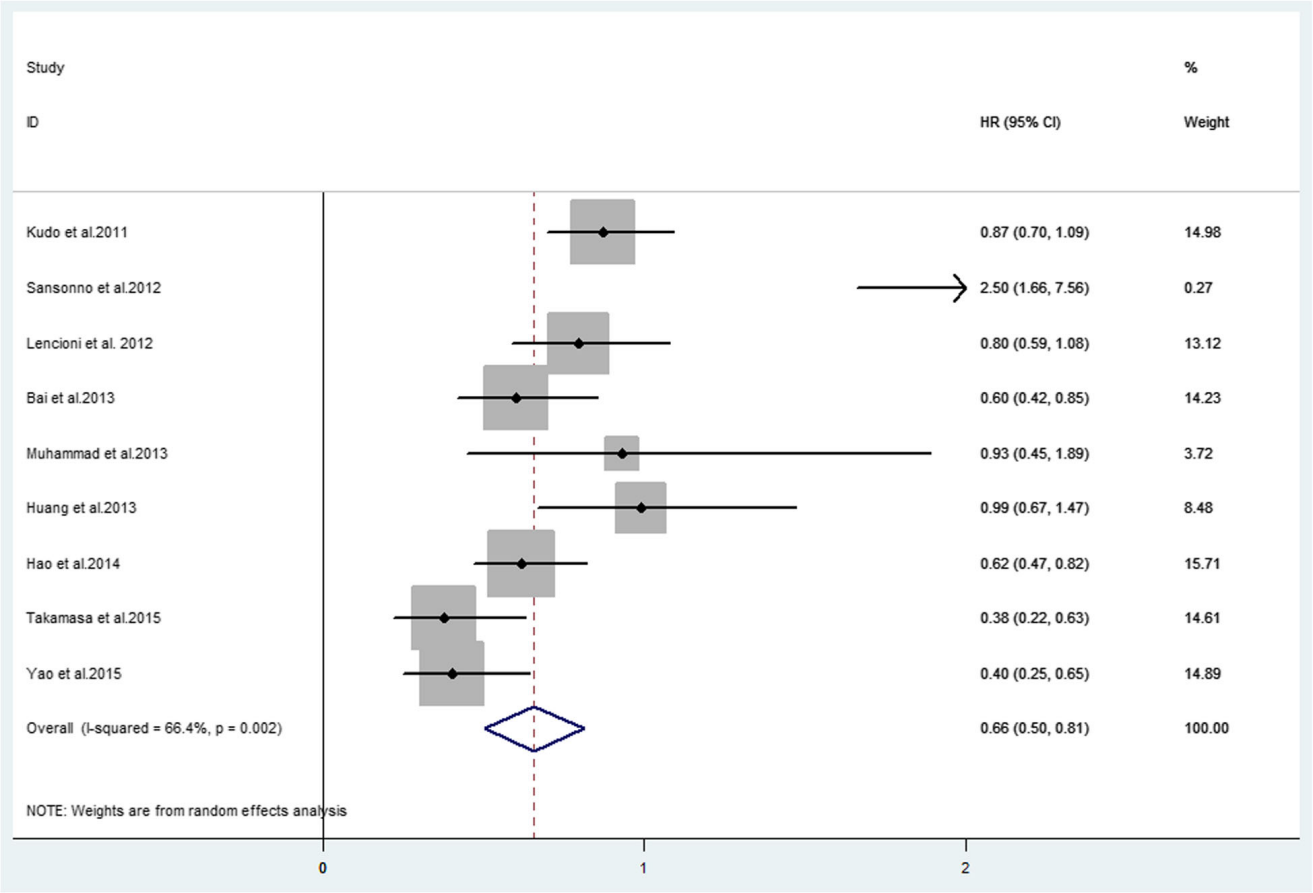
Forest plot of TTP outcome between TACE alone and combination therapy for unresectable HCC

**Fig. 3:**
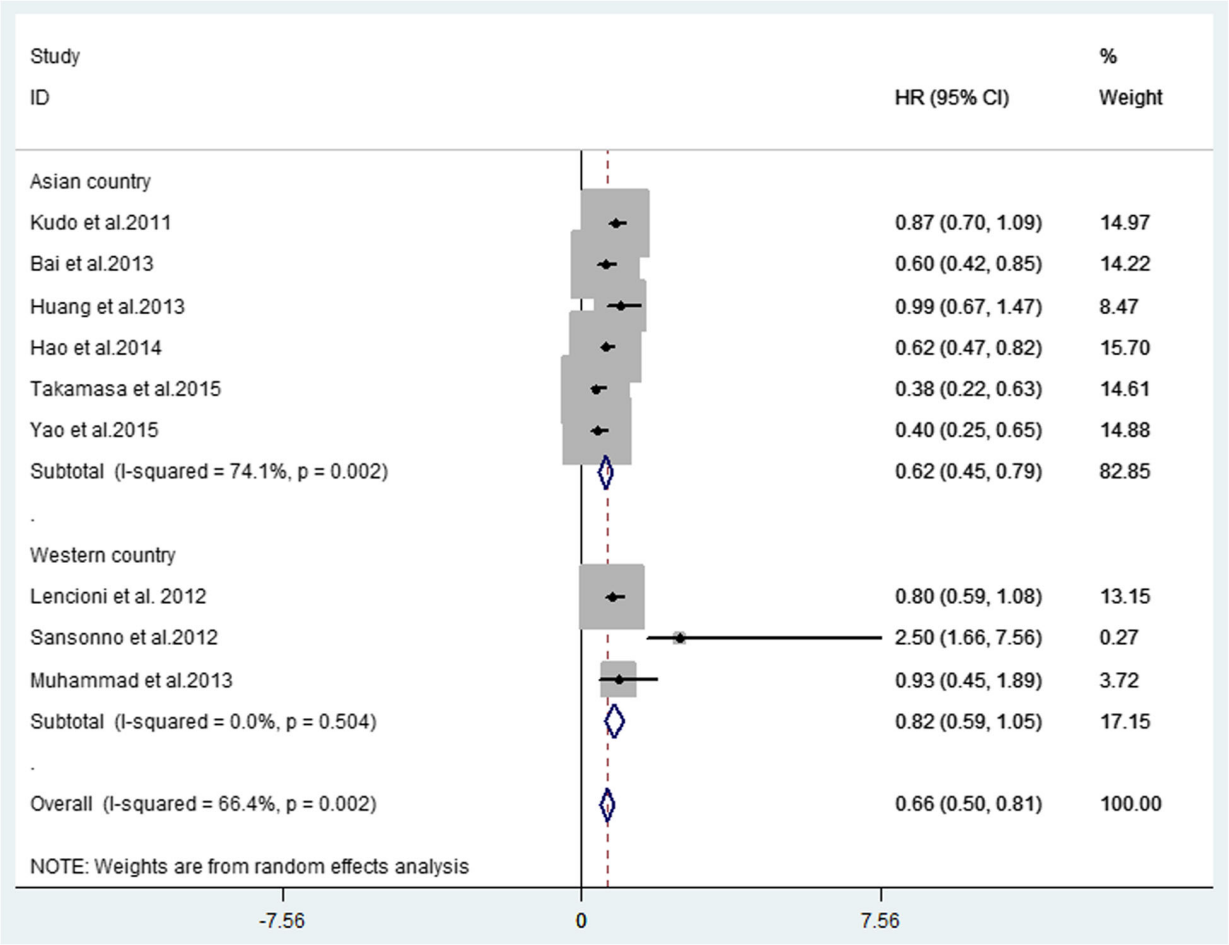
Subgroup analysis of region of TTP outcome between TACE alone and combination therapy

Ten studies provided TTP with a median ranging from 2.6 to 10.2 months. Nine studies provided available HR for TTP (Table 3). The forest plot showed that the overall HR for TTP was 0.66 (95% CI 0.50–0.81, *P* = 0.002), indicating that combination therapy significantly prolonged TTP. The analysis was performed in a random effect model and the I^2^ was 66.4% (Fig. 2). To minimize heterogeneity, TTP in Asia-Pacific and Western studies were separately analysed by the sub-analysis of forest plots. The forest plot showed that the HR for TTP in Asian countries was 0.62 (95% CI 0.45–0.79, *P* = 0.002) and was 0.82 (95% CI 0.59–1.05, *P* = 0.504) in western countries (Fig. 3). These data may indicate positive TTP outcome of statistical significance in Asian countries. Regions may show differences in survival outcome through various factors.

**Table 3:** Median TTP, HR and 95%CIs between combination therapy group and TACE alone group

Authors (year)	Combination group (95% CI)/months	TACE alone group (95% CI)/months	HR (95% CI)
Kudo et al. (2011) [15]	5.4(3.8–7.2)	3.7 (3.5–4.0)	0.87(0.70–1.09)
Sansonno et al.(2012) [44]	9.2	4.9	2.5(1.66–7.56)
Lencioni et al. (2012) [10]	5.6	5.5	0.797 (0.588–1.08)
Bai et al. (2013) [46]	6.3	4.3	0.6 (0.422–0.853)
Muhammad et al. (2013) [47]	NA	NA	0.93 (0.45–1.89)
Huang et al. (2013) [48]	5.4	3.7	0.99 (0.67–1.47)
Hu et al. (2014) [14]	2.6	1.9	0.62 (0.47–0.82)
Ohki et al. (2015) [6]	6.3	3.5	0.38 (0.22–0.63)
Yao et al. (2015) [12]	10.2	6.7	0.403 (0.251–0.646)
Zhang et al. (2016) [49]	4.9 (3.7–6.0)	2.4 (1.3–3.4)	NA

*Abbreviations*: *TTP* time to progression, *HR* hazard ratio, *95%CIs* 95% confidence intervals, *NA* not available

##### OS

**Fig. 4:**
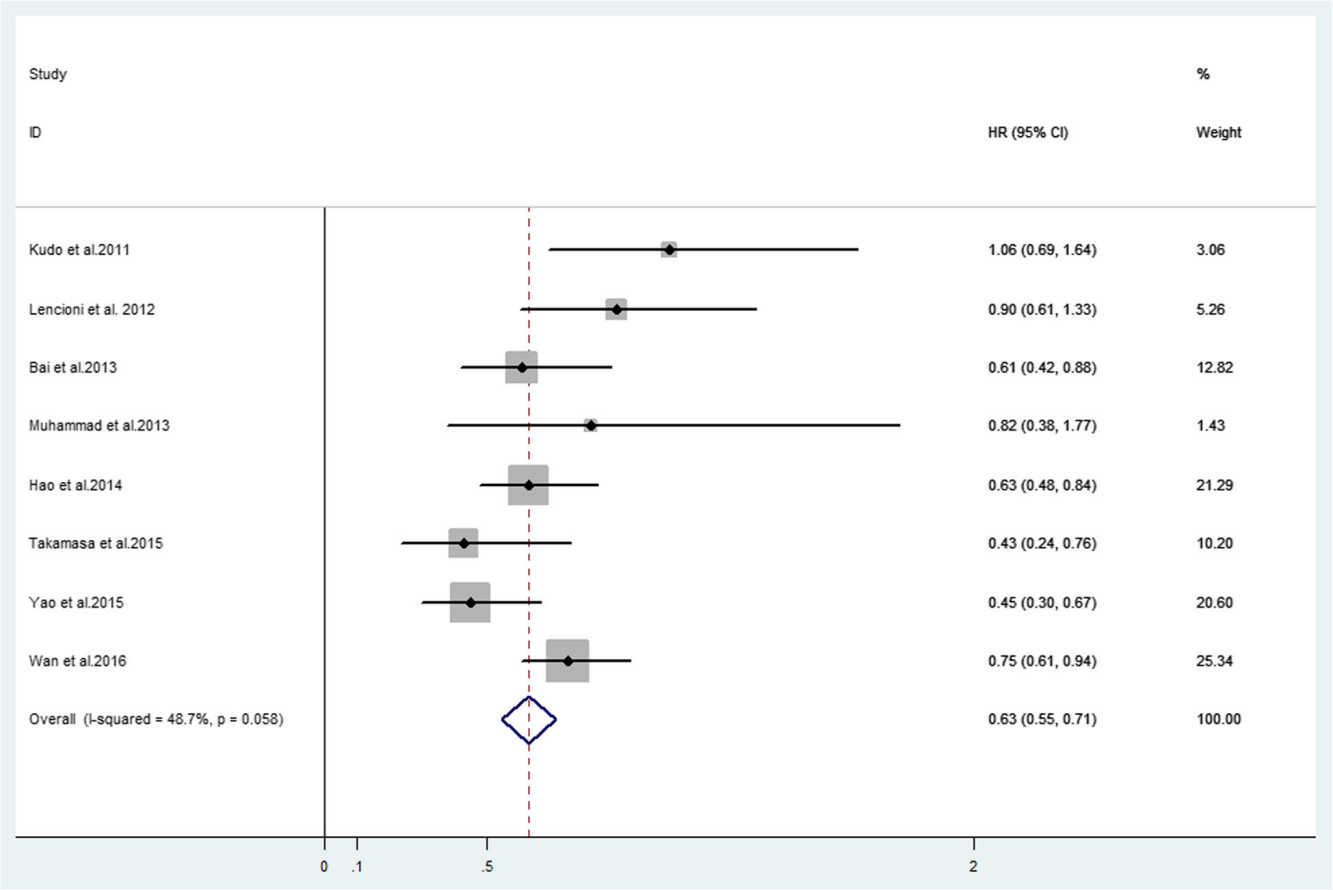
Forest plot of OS outcome between TACE alone and combination therapy for unresectable HCC

**Fig. 5:**
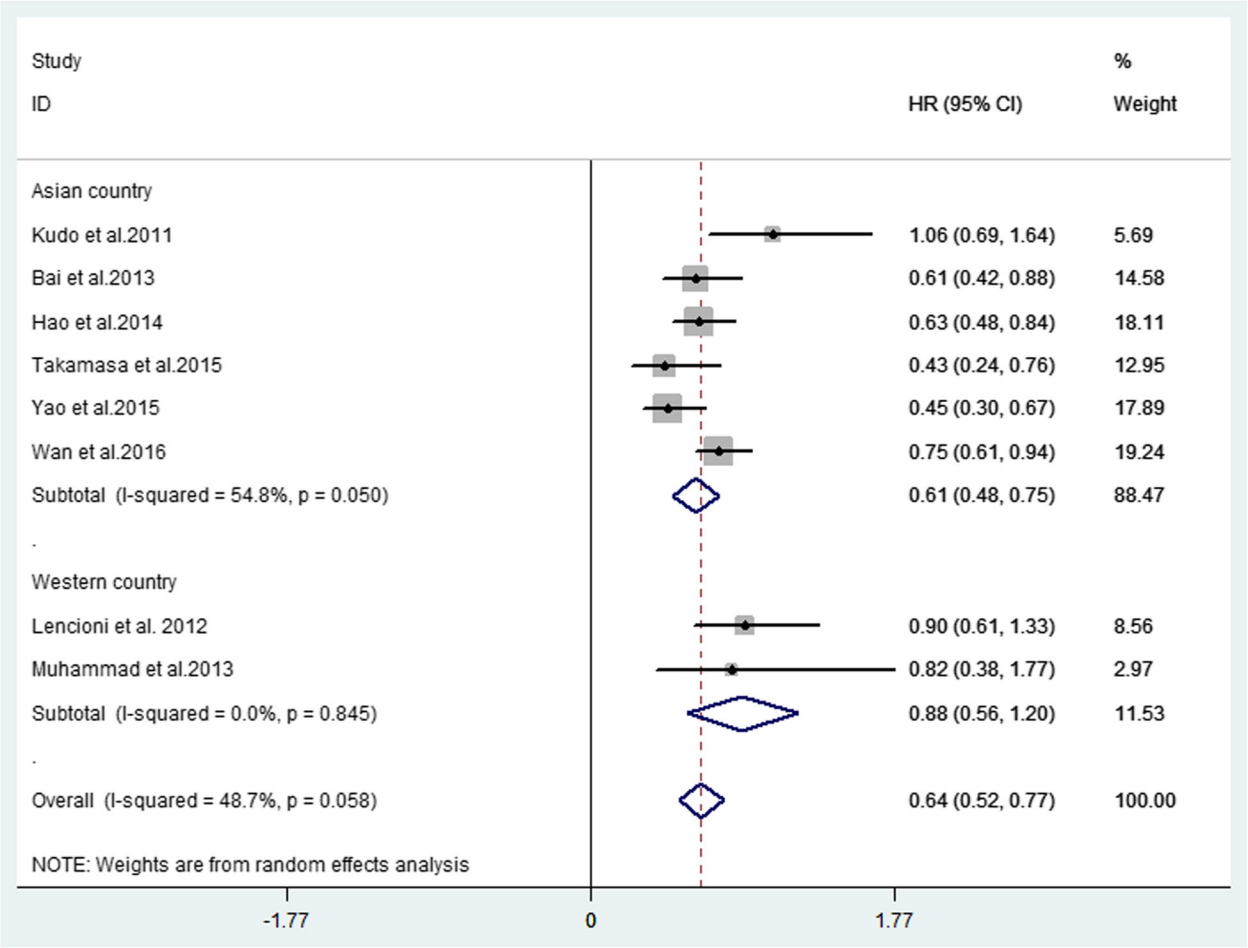
Subgroup analysis of region of OS outcome between TACE alone and combination therapy

Ten studies reported median OS ranging from 7.0 to 29.7 months, while HR of OS was available in 8 studies (Table 4). The forest plot indicated that the overall HR for OS was 0.63 (95% CI 0.55–0.71, *P* = 0.058), suggesting that combination therapy may not significantly improve OS. The analysis was performed in a fixed effect model and the I^2^ was 48.7% (Fig. 4). The subgroup analysis according to different region was also performed, and the HR for OS was 0.61 (95% CI 0.48–0.75, *P* = 0.050) in Asian countries and was 0.88 (95% CI 0.56–1.20, *P* = 0.845) in western countries (Fig. 5), without statistical significance across different regions.

**Table 4:** Median OS, HR and 95%CIs between intervention and contrast group

Authors (year)	Combination group (95% CI)/months	TACE alone group (95% CI)/months	HR (95% CI)
Kudo et al. (2011) [15]	29.7 (28.6-NA)	NA	1.06 (0.69–1.64)
Lencioni et al. (2012) [10]	NA	NA	0.898 (0.606–1.33)
Qu et al. (2012) [45]	27 (21.9–32.1)	17 (8.9–25.0)	NA
Bai et al. (2013) [46]	7.5	5.1	0.61 (0.423–0.884)
Muhammad et al. (2013) [47]	20.6 (13.4–38.4)	18.3 (11.8–32.9)	0.82 (0.38–1.77)
Hu et al. (2014) [14]	7.0	4.9	0.63 (0.48–0.84)
Ohki et al. (2015) [6]	28.7	15.6	0.43 (0.24–0.76)
Yao et al. (2015) [12]	21.7	11.5	0.449 (0.302–0.668)
Wan et al.(2016) [50]	20.23	13.97	0.75 (0.61–0.94)
Zhang et al. (2016) [49]	14.9 (6.8–23.0)	6.1 (4.0–8.1)	NA
Varghese et al. (2017) [13]	BCLC-B = 16 (12.9–19.1)	BCLC-B = 9 (6.3–11.7)	BCLC-B:NA
	BCLC-C = 9 (6.8–11.2)	BCLC-C = 4(3–5)	BCLC-C:NA

*Abbreviations*: *OS* overall survival, *HR* hazard ratio, *95%CI* 95% confidence intervals, *NA* not available

#### Relationship between aetiology and survival outcome

Four studies provided HR of aetiology for OS, and 3 studies provided HR for TTP (Table 5). Using random effect models, the forest plots indicated that the overall HR of aetiology for OS was 1.10 (0.78–1.41, *P* = 0.888) (Additional file 3: Figure S1), and the overall HR for TTP was 0.88 (0.72–1.05, *P* = 0.565) (Additional file 4: Figure S2). We may deduce that the aetiology of HCC might not have significant influence on survival outcome.

**Table 5:** The HR of etiology in the studies

Authors (year)	Study design	Aetiology	Endpoint	HR
Kudo et al. (2011) [15]	RCT trial	HBV = 20%	TTP	0.81(0.62–1.07)
		HCV = 60%		
Bai et al. (2013) [46]	Comparative study	HBV = 87.9%	OS	1.01(0.60–1.71)
		HCV = 4.9%		
Muhammad et al. (2013) [47]	Comparative study	ST:HCV = 69%	OS	1.04(0.66–1.63)
		DT:HCV = 93%		
Zhao et al. (2013) [41]	Non-comparative study	HBV = 80%	OS	1.372(0.773–2.437)
		HCV = 5%		
Hu et al. (2014) [14]	Comparative study	ST:B = 82.9%	TTP	1.01(0.76–1.34)
		T:B = 79.8%		
Yao et al. (2016) [12]	Comparative study	ST:HBV = 84%	OS	1.228(0.593–2.540)
		T:HBV = 83%	TTP	0.878(0.494–1.561)

*Abbreviations: HR* hazard ratio, *OS* overall survival, *TTP* time to progression, *RCT* randomized controlled trials, *ST* sorafenib plus TACE, *DT* DEB –TACE

### Adverse events

AEs of combination therapy included fatigue, diarrhoea, nausea, hand-foot skin reaction (HFSR), haematological events, alopecia, hepatotoxicity, hypertension and rash (Additional file 5: Table S3). Among these, the incidence of HFSR was highest. Most patients experienced at least one type of sorafenib-related AE during drug administration. Most AEs were mild to moderate and could be controlled through appropriate management, including temporary dose reduction or another syndrome-relieving treatment. The incidence of severe AEs, such as hepatic failure or gastrointestinal haemorrhage, was very low. No treatment-related deaths and disabilities occurred in these studies.

## Discussion

Several clinical trials have been conducted to evaluate the efficacy of combination therapy. Our systematic review and meta-analysis collected the updated studies that evaluated the efficacy of combination therapy for unresectable HCC. The studies were published during the past 8 years, including comparative and non-comparative trials. The comprehensive analysis of 27 studies indicated that combination therapy may have significant superiority over TACE mono-therapy in terms of TTP but not OS.

As the first globally randomized controlled trial with a relatively large sample size, the SPACE trial (sorafenib or placebo in combination with TACE for intermediate-stage HCC) conducted by Lencioni et al. showed no significant difference of TTP between the combination therapy group and the TACE alone group [10]. Later, many clinical trials conducted in different countries also evaluated the efficacy of combination treatment, and most reported findings that combination therapy was more effective than mono-therapy in terms of TTP. Among 14 comparative studies that we analysed, most studies concluded that, compared with TACE alone, combination treatment with TIPS followed by sorafenib increased the TTP in patients unresponsive to TACE [11–14].

Kudo et al. found the outcomes of clinical trials varied across different races and regions. For Japanese patients, the HR for TTP was 0.94 (95% CI, 0.75–1.19), while for Korean patients it was 0.38 (95% CI, 0.18–0.81), suggesting that the Korean patients may benefit more from combination therapy than Japanese patients [15]. Compared with other Asian countries, Japanese HCC patients had higher HCV infection rates. However, our analysis between aetiology and survival showed no significant difference. Studies have shown that the mechanism of HCC caused by HBV and HCV is different [16], and pathological manifestations and gene expression differ between HBV- and HCV-related HCC [17, 18]. In terms of tumour survival and prognosis, some studies found significantly better survival and smaller recurrence rates in HCV-related HCC than with HBV-related HCC [19, 20]. In contrast, other studies showed that the prognosis of HCV-related HCC patients was worse than that of HBV-related HCC patients [21]. This might be a potential reason for our negative finding, since the proportion of HCV-related HCC patients in the 27 studies included in this analysis was small.

The survival rate in the Asian-Pacific region was lower than that of European countries. In particular, the mortality rate of Chinese patients was higher than the average value of other regions in the world. Our analysis of regions showed that the TTP outcome in the Asian group was positive, while the European group returned a negative result. In another analysis, both groups showed a negative OS outcome. However, regions show differences through many factors. Take treatment procedure for example; in SPACE trials, there was a greater improvement in TTP and OS HRs in patients from Asian countries than from non-Asian countries. Because non-Asian patients in the sorafenib arm discontinued TACE treatments earlier and had a shorter duration of sorafenib, both factors may have contributed to the outcome difference and may have caused bias [10]. Well-designed studies, regular drug administration and good control of confounding factors are needed to reflect the real efficacy of combination therapy.

C-TACE is performed by the injection of a mixture of a chemotherapeutic drugs and lipiodol, which block feeding vessels, and thus cause tumour necrosis [22]. DEB-TACE releases chemotherapeutic agents from micro-beads, facilitating further, more effective and more focused embolization [23, 24]. However, compared with C-TACE, it appears that DEB-TACE shows similar clinical outcomes with fewer adverse events. In terms of efficacy, whether DEB-TACE is superior to C-TACE remains debatable [25, 26].

Although there were no positive findings regarding OS in the meta-analysis, this does not necessarily suggest that combination therapy was not futile for improving the survival time of HCC patients. Many clinical trials also have shown that combination therapy can prolong OS [4, 6, 11–13]. The heterogeneity of patients’ physical conditions may be the primary factor affecting OS, as candidate selection may make a difference. Various study designs, including treatment procedure, number of TACE and duration of sorafenib administration might also have an effect on the outcome. In this case, reasonable study design, including proper candidate selection and appropriate treatment administration, are of great concern [6].

Lead time bias is another factor that may have impact on survival outcome. Lead time means the interval by which the disease was diagnosed by screening in advance [27]. It might create bias in observational studies of screening efficacy and may affect the comparison of overall survival among various studies [28]. However, the BCLC staging system might have made a relatively clear classification for HCC. Currently, most clinical trial designs use inclusion criteria based on BCLC stage, possibly helping to reduce this bias to some degree.

Some studies that included HCC patients with portal vein invasion have shown that combination therapy was more effective than TACE alone in terms of TTP and OS [29, 30]. However, other studies suggested negative efficacy that combination therapy brought for HCC patients with portal vein invasion [7, 14]. The extent of portal vein invasion may make difference to the survival effects. Moreover, promising OS of combined therapy with worse baseline condition may be attributed to incorporate administered systemic therapy and loco-regional treatments [30]. Another study focusing on combination efficacy between elderly and non-elderly patients concluded that age was not a prognostic factor for treatment outcome in advanced HCC patients [11, 31].

In terms of AEs, the study by Yao et al. found that combination therapy induced greater AEs than did TACE mono-therapy [32]. According to the final analysis of the START trials, combination therapy did not appear to lead to worse AEs. Moreover, the presence of some AEs such as HFSR indicated positive correlation with anti-tumour efficacy [15].

The major potential limitations of the present study are as follows: First, the number of studies included in this meta-analysis was relatively large, with half being non-comparative — the heterogeneity of available data from these studies was correspondingly substantial. The funnel plots also showed potential publication bias. Second, only several studies conducted OS and TTP analysis. The detailed information available for meta-analysis was limited. Third, the retrospective nature, small sample size, non-randomized study design and the various treatment procedures may increase the uncertainty of the conclusions.

## Conclusions

As a meta-analysis which included a large number of studies, overall results of this systematic review and meta-analysis suggest that the combination of sorafenib plus TACE was superior to TACE alone in terms of TTP but not OS. Nevertheless, combination therapy is still effective and promising. This study not only analysed the relationship between combination therapy and survival efficacy to clarify this controversial issue, but also provided conclusions that aetiological differences may not influence survival outcomes. Separated regions analysis contributed to less heterogeneity while other similar studies currently lack such analysis. In the future, well-designed, randomized-controlled, prospective trials with optimized study designs and large sample sizes are required.

## Additional files

Additional file 1: **Table S1.** Tumor response criteria, DCR, TTP and OS in 13 non-comparative studies. (DOCX 21 kb)

Additional file 2: **Table S2.** DCR in 5 comparative studies. (DOCX 19 kb)

Additional file 3: **Figure S1.** Forest plot of TTP outcome about the relationship between etiology and treatment outcome. (TIF 1710 kb)

Additional file 4: **Figure S2.** Forest plot of OS outcome about the relationship between etiology and treatment outcome. (TIF 1717 kb)

Additional file 5: **Table S3.** The AEs occurred during combination therapy in 13 non-comparative studies. (DOCX 21 kb)

## Abbreviations

95% CIs: 95% confidence intervals
AEs: Adverse events
BCLC: Barcelona Clinic Liver Cancer
c-TACE: Conventional transarterial chemoembolization
DCR: Disease control rate
DEB-TACE: Transarterial chemoembolization with drug-eluting beads
HBV: Hepatitis B virus
HCC: Hepatocellular carcinoma
HCV: Hepatitis C virus
HFSR: Hand-foot skin reaction
HR: The hazard ratio
MINORS: Methodological index for non-randomized studies
mRECIST: Modified RECIST
OS: Overall survival
PDGF: Platelet-derived growth factor
RECIST: Response Evaluation in Solid Tumors
TACE: Transarterial chemoembolization
TTP: Time to progression
VEGF: Vascular endothelial growth factor
