# Supplementary material for: Transarterial chemoembolization plus sorafenib for the management of unresectable hepatocellular carcinoma: a systematic review and meta-analysis
Source: BMC Gastroenterol. 2018 Sep 4;18:138. doi: 10.1186/s12876-018-0849-0 (PMC6124009; doi:10.1186/s12876-018-0849-0)
Supplement: Supplementary file 1 — Table S1. Tumor response criteria, DCR, TTP and OS in 13 non-comparative studies. (DOCX 21 kb) [file 12876_2018_849_MOESM1_ESM.docx]

| Table S1. Tumor Response criteria, DCR, TTP and OS in 13 non-comparative studies. | | | | |
| --- | --- | --- | --- | --- |
| Authors | **Response criteria** | **DCR (%)** | **Median TTP/ months** | **Median OS /months** |
| Erhardt et al. | RECIST | 18.4 | NA | NA |
| Dufour et al. | NA | NA | NA | NA |
| Cabrera et al. | mRECIST | 68 | NA | 18.5(95%CI 16.1-20.9) |
| Lee et al. | mRECIST | 76 | NA | NA |
| Pawlik et al. | RECIST | 95 | NA | NA |
| Chung et al. | mRECIST | 91.2 | 9 | NA |
| Park et al. | RECIST | 84 | 7.1 (95%CI 4.8–7.5) | NA |
| Sieghart et al. | mRECIST | 80 | NA | NA |
| Zhao et al. | RECIST | 86 | NA | 12(95%CI 10.1-13.9) |
| Pan et al. | RECIST | 80.5 | 7 (95%CI 5.7-8.3) | 13 (95%CI 10.4-15.6) |
| Chao et al. | mRECIST | 93.7 | 13.8 (95%CI 11.3-16.4) | NA |
| Yao et al. | mRECIST | NA | 7 | NA |
| Cosgrove et al. | RECIST | 94 | 13.9 (95%CI 8.5-27.6) | 20.4 |
| Abbreviations: TTP, time to progression; OS, overall survival; DCR, disease control rate; RECIST, response evaluation in solid tumors; mRECIST, modified RECIST; NA, not available. | | | | |
