# Supplementary material for: Transarterial chemoembolization plus sorafenib for the management of unresectable hepatocellular carcinoma: a systematic review and meta-analysis
Source: BMC Gastroenterol. 2018 Sep 4;18:138. doi: 10.1186/s12876-018-0849-0 (PMC6124009; doi:10.1186/s12876-018-0849-0)
Supplement: Supplementary file 2 — Table S2. DCR in 5 comparative studies. (DOCX 19 kb) [file 12876_2018_849_MOESM2_ESM.docx]

| Table S2. DCR in 5 comparative studies. | | |
| --- | --- | --- |
| Authors(year) | **Combination group** **(%)** | **TACE alone group (%)** |
| Lencioni et al. (2012) | 89.2 | 76.1 |
| Bai et al. (2013) | 58.5 | 44.5 |
| Yao et al. (2015) | 32 | 24 |
| Zhang et al. (2016) | 80 | 43.3 |
| Varghese et al. (2017) | BCLC-B=97.2  BCLC-C=97.2 | BCLC-B=70  BCLC-C=16.7 |
| Abbreviations: DCR, disease control rate; BCLC, The Barcelona Clinic Liver Cancer. | | |
