# Supplementary figures and images for: Transarterial chemoembolization plus sorafenib for the management of unresectable hepatocellular carcinoma: a systematic review and meta-analysis
Source: BMC Gastroenterol. 2018 Sep 4;18:138. doi: 10.1186/s12876-018-0849-0 (PMC6124009; doi:10.1186/s12876-018-0849-0)

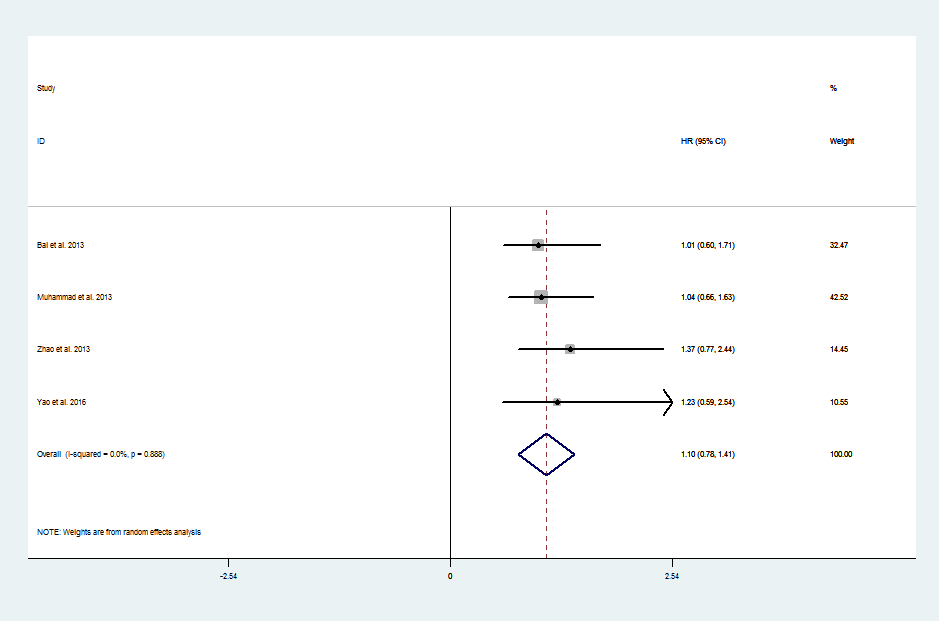

Supplement: Supplementary file 3 — Figure S1. Forest plot of TTP outcome about the relationship between etiology and treatment outcome. (TIF 1710 kb) [file 12876_2018_849_MOESM3_ESM.tif]

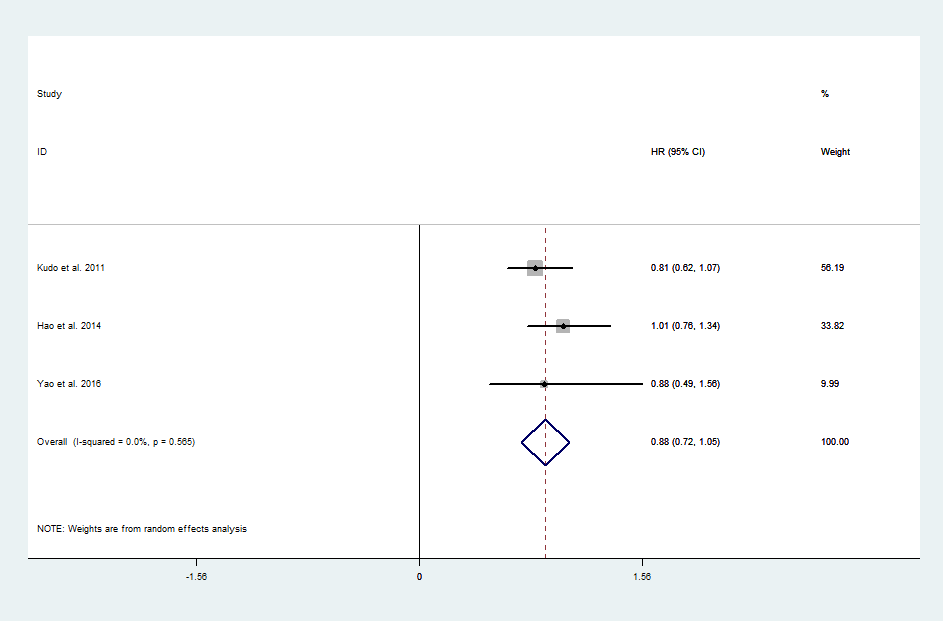

Supplement: Supplementary file 4 — Figure S2. Forest plot of OS outcome about the relationship between etiology and treatment outcome. (TIF 1717 kb) [file 12876_2018_849_MOESM4_ESM.tif]
