# Supplementary material for: Transarterial chemoembolization plus sorafenib for the management of unresectable hepatocellular carcinoma: a systematic review and meta-analysis
Source: BMC Gastroenterol. 2018 Sep 4;18:138. doi: 10.1186/s12876-018-0849-0 (PMC6124009; doi:10.1186/s12876-018-0849-0)
Supplement: Supplementary file 5 — Table S3. The AEs occurred during combination therapy in 13 non-comparative studies. (DOCX 21 kb) [file 12876_2018_849_MOESM5_ESM.docx]

| Table S3. The AEs occurred during combination therapy in 13 non-comparative studies. | | | | | | | | | |
| --- | --- | --- | --- | --- | --- | --- | --- | --- | --- |
| Authors | **HFSR (%)** | **Diarrhea (%)** | **Fatigue (%)** | **Hematological events (%)** | **Alopecia (%)** | **Hepatotoxicity (%)** | **Hypertension (%)** | **Nausea (%)** | **Rash/Desquamation (%)** |
| Erhardt et al. | 5 | NA | 5 | NA | NA | 5 | NA | NA | NA |
| Dufour et al. | 21 | 50 | NA | 14 | NA | 20 | NA | NA | NA |
| Cabrera et al. | 51.1 | 42.5 | 51 | 10.6 | 4.3 | 23 | 19 | 14.9 | 15 |
| Lee et al. | NA | NA | NA | NA | NA | NA | NA | NA | NA |
| Pawlik et al. | 40 | 33 | 80 | 38 | 35 | 55 | NA | NA | NA |
| Chung et al. | 3.4 | 18.4 | 8.2 | 6.8 | 25.9 | 4 | 8.8 | 19 | 28 |
| Park et al. | 74 | 48 | 10 | 14 | 24 | 36 | NA | 54 | 12 |
| Sieghart et al | 4 | 50 | 95 | 50 | 75 | 75 | 17 | 50 | 20 |
| Zhao et al. | 44 | 50 | 33 | NA | 4 | 4 | NA | 1 | 39 |
| Pan et al. | 68.3 | 53.7 | 22 | NA | 19.5 | NA | 9.8 | 19.5 | 22 |
| Chao et al. | NA | 31.3 | 11.5 | 7.3 | NA | 10.4 | 21.4 | 25 | 31.8 |
| Yao et al. | 58 | 40 | 54 | NA | NA | NA | 38 | NA | NA |
| Cosgrove et al. | 46 | 38 | 84 | 90 | NA | NA | 14 | 48 | 52 |
| Abbreviations: AEs, adverse events; HFSR, hand foot skin reaction; NA, not available. | | | | | | | | | |
